# Singular Case, of Scarlatina Form, Measly and Variolous Disease, Combined in the Same Patient

**Published:** 1872-04-01

**Authors:** Thos. Bevan


### SINGULAR CASE, OF SCARLATINA FORM, MEASLY AND VARIOLOUS DISEASE, COMBINED IN THE SAME PATIENT.

BY THOS. BEVAN, M.D.

Mr. B——e, of No. 15 Harmon Court, Chicago, began to complain on Friday, 23d of February, and grew worse on Saturday. There was general soreness, headache, fever, intense bronchial and nasal catarrh, and pain over whole body, most marked in head and back. He took some domestic remedies, suggested by non-professional friends, and felt somewhat improved, but not fully relieved. Mr. B——e had about the 14th of the month been on a night journey in a sleeping car, and thought he took cold, as he had not been quite well after that date, until taken down. Was called on the 27th, as medical adviser, for the first time; pulse 120 to 125 and 130 per minute; skin hot and dry; headache; sibilant, dry, and moist rales over both sides of the chest; cough incessant, tearing and painful; eyes congested, and head turgescent; back and limbs the seat of soreness and pain; great restlessness; nervous agitation.

Prescribed saline laxative and syr. liquor. comp., together with Dover powd., occasionally and at night, and sinapism over chest. 28th. Cough less frequent, expectoration free. expressed himself better, went on improving to Friday, March 1.

March 2. Slight delirium, fearful headache, backache, herpetic eruption about mouth, one papular point, tending to pustulation, on right malar surface, one on forehead, impetigenoid (accumulated after a day or two, without depressed center), two or three on thighs, some vesicles, small and not unlike sudamina. March 2, p. m. After 10 days' illness, papular eruption on bald space on cranium, red efflorescence, and decided swelling of whole head. Diagnosis—Varioloid, with some uncertainty about its form. Reported as such. Papule, for twenty or twenty-four hours, disappears on pressure; then a small drop of blood is extravasated at the site of each papule; not capable of being made to disappear by pressure at all. Patient in a fearfully agitated state; delirium when he dozes, but sleeps little or none; eruption extends to whole body, everywhere hemorrhagic. Probable diagnosis and prognosis—black, hemorrhagic small pox, and death. Treatment, simple diluents; neut. mixt. Dover powder; disinfection of the air of room every two hours, with the spray of carbolic acid and chlorinated soda, from atomizer, together with the use of chloride of lime, carbolic acid, and thorough ventilation. A temperature of 65° in the room ordered night and day; for heat of head, sponging with tepid water. Isolation had of course been practiced from first pimple. Whiskey, egg and milk were used, for prostration was pronounced; also, beef essence and nourishing fluids of all kinds. Stomach irritable—lime water.

*Presto!* On the 5th, amelioration of all symptoms—cough, fever, delirium, headache —*all* better; papule still hemorrhagic, but color gradually fading.

Gradual improvement from this on to 14th, when after alkaline baths and thorough disinfection and destruction of all possible fomites had been accomplished, isolation was discontinued. This was just about one month from possible sleeping car exposure. To-day, March 18, saw him; had been out; some bronchial irritation still, and a little intestinal mucous irritation. Now, wise reader, what in the name of the divine Esculapius has been the matter? What was the *materia morbi* back of this most anomalous eruption? My belief is that it was modified black or hemorrhagic small pox, and I acted in the interest of the exposed as if such had been the case. How does it fit this description of that disease, from Trousseau's Clin. Med., see p. 81–2, Vol. 2d: "It is more common to meet with anomalous cutaneous eruptions, *according to the prevailing epidemic constitution*, in modified than in unmodified small pox." (The patient had been vaccinated and re-vaccinated.) "They appear the day before, or simultaneously with the pustules. Sometimes they so much simulate as to be mistaken for *the eruption of measles, even when looked at closely*." (The italics are my own.) "Still more do they resemble the exanthema of scarlatina." (My case did this exactly and perfectly.) "They do not disappear on pressure with finger. These hemorrhagic scarlatina-form eruptions, which in natural small pox constitute an alarming symptom, do not lead to unfavorable prognosis in modified small pox. They are sometimes on groin, thighs, abdomen; sometimes more generally diffused. The condition of the patient is then apparently more serious," etc., etc.

Now, these are my reasons for thinking the case small pox. There was and is an epidemic variolous constitution prevailing. There was not and is not such epidemic constitution of morbilli, or measles. I have seen hemorrhagic measles, but Sydenham and others say it, also, is a very fatal disease, though the cases I saw during the war did not die, except when complicated with serious pulmonary phlegmasia. Such cases are exceedingly interesting as studies of anomalous morbid states, and they are exceedingly well calculated to keep the medical attendant wide awake; for between the asses who want everything decided in a minute, and the long talkers who wish to know all about it anyway, the attendant, if a little nervous, sounds all the notes in the gamut of study, from ebulant profanity, if he isn't pious, to the quiet, studious mood of methodic observation.

That what is known as the hemorrhagic diathesis exists, to a certain extent, in all such cases, I think is to be admitted, but the usual signs of diminished vigor, from any general or traceable causes, are not always present.

Mr. B——e was a robust man of 35 years of age.

